# Walnut Phenolic Extract and Its Bioactive Compounds Suppress Colon Cancer Cell Growth by Regulating Colon Cancer Stemness

**DOI:** 10.3390/nu8070439

**Published:** 2016-07-21

**Authors:** Jisoo Lee, Yoo-Sun Kim, JaeHwan Lee, Seung Chul Heo, Kook Lae Lee, Sang-Woon Choi, Yuri Kim

**Affiliations:** 1 Department of Nutritional Science and Food Management, Ewha Womans University, Seoul 03760, Korea; dlwltn233@naver.com (J.L.); tidygirlss@naver.com (Y.-S.K.); 2 Department of Food Science and Biotechnology, Sungkyunkwan University, Suwon 16419, Korea; s3hun@skku.edu; 3 Department of Surgery, Seoul Metropolitan Government-Seoul National University Boramae Medical Center, Seoul 07061, Korea; heosc3@brmh.org; 4 Department of Internal Medicine, Seoul National University Boramae Hospital, Seoul National University College of Medicine, Seoul 07061, Korea; kllee@brmh.org; 5 Chaum Life Center, CHA University, Seoul 06062, Korea; sangwoon.choi@gmail.com

**Keywords:** walnut phenolic extract, bioactive compounds, colon cancer, cancer stem cells, self-renewal capacity

## Abstract

Walnut has been known for its health benefits, including anti-cardiovascular disease and anti-oxidative properties. However, there is limited evidence elucidating its effects on cancer stem cells (CSCs) which represent a small subset of cancer cells that provide resistance against chemotherapy. This study aimed to evaluate the anti-CSCs potential of walnut phenolic extract (WPE) and its bioactive compounds, including (+)-catechin, chlorogenic acid, ellagic acid, and gallic acid. In the present study, CD133^+^CD44^+^ cells were isolated from HCT116 cells using fluorescence-activated cell sorting (FACS) and then treated with WPE. As a result, survival of the CD133^+^CD44^+^ HCT116 cells was inhibited and cell differentiation was induced by WPE. In addition, WPE down-regulated the CSC markers, CD133, CD44, DLK1, and Notch1, as well as the β-catenin/p-GSK3β signaling pathway. WPE suppressed the self-renewal capacity of CSCs. Furthermore, the WPE exhibited stronger anti-CSC effects than its individual bioactive compounds. Finally, the WPE inhibited specific CSC markers in primary colon cancer cells isolated from primary colon tumor. These results suggest that WPE can suppress colon cancer by regulating the characteristics of colon CSCs.

## 1. Introduction

Colorectal cancer (CRC) is the third most commonly diagnosed cancer and the third leading cause of cancer deaths in the United States for both men and women. In 2015, it was predicted that 132,700 people would be diagnosed with CRC, while 49,700 people would succumb to CRC in the U.S. [1]. Despite advances in CRC screening, as well as the use of surgical resection and chemotherapeutic drugs, 50% of CRC patients experience recurrence. These higher recurrence rates for CRC are consistent with characteristics of cancer stem cells (CSCs), including self-renewal capacity, differentiation, and tumorigenicity. In addition, CSCs can exhibit enhanced proliferation and invasion properties, and can thus induce the tumor relapse and enhance drug-resistance in tumors [2,3]. Therefore, targeting of CSCs is crucial to a successful therapeutic strategy for CRC.

CSCs have been identified based on the expression of specific cell surface markers. Among these, CD133, CD44, aldehyde dehydrogenase (ALDH1), and CD24 are the most widely used for the isolation of CSCs, and they are also expressed in CRC [4]. Drosophila delta-like 1 homolog (DLK1) is an epidermal growth factor (EGF)-like protein that has a role in the differentiation of both adipocytes and hematopoietic stem cells [5,6]. Notch1 homolog 1 (Notch1) is a single trans-membrane receptor that contributes to the maintenance of stem cells, cell proliferation, and apoptosis [7]. Wnt/β-catenin signaling, which represents a key pathway in embryonic development and self-renewal, also has a role in CRC development [4]. Finally, β-catenin is phosphorylated by a protein complex of glycogen synthase kinase 3β (GSK3β), which promotes ubiquitination and degradation by the proteasome [8].

Walnuts (*Juglans regia*) have been reported to have a number of health benefits [9,10], including anti-cardiovascular disease, anti-oxidative properties, and cancer preventive properties [11,12,13]. In addition, several epidemiological studies have shown an inverse association between the intake of nuts and seeds and the risk of CRC [14,15], while the consumption of walnuts has been associated with a reduced growth rate of breast cancer and CRC in xenograft mouse models [16,17,18]. Walnuts are rich in the essential fatty acids, vitamin E, and folate [13]. It was reported that walnut lipid extract which contains high concentrations of α-linoleic acid, α-linolenic, oleic acid, and γ-tocopherol, was able to suppress colon CSCs by regulating their self-renewal capacity [19]. They also contain large amounts of polyphenols, including ellagic acid, gallic acid, and quercetin [9,20]. Furthermore, polyphenols have been found to exhibit anti-cancer effects [21,22].

However, despite the health benefits that are associated with walnuts, the potential mechanism(s) underlying the anti-CSC effects of walnut phenolic extract (WPE), as well as the phenolic bioactive compounds of WPE in CRC, have not been investigated. Therefore, we examined the effects of WPE on the stemness of CSCs using CD133^+^CD44^+^ HCT116 cells, and found that WPE and its bioactive compounds induced differentiation and reduced CSC markers and self-renewal capacity. These results support a rationale for further research of the effects of WPE on prevention of occurrence and recurrence of human malignant CRC.

## 2. Materials and Methods

### 2.1. Preparation of WPE

WPE from English walnuts (*J. regia*, California Walnut Commission) was prepared according to a previously described methanolic extraction method [9]. Briefly, after the walnuts were frozen for 24 h, the shelled kernels were finely ground and immersed in a solution of 75% acetone containing 526 μm/L sodium metabisulfite. The solution was subsequently purged with *N*_2_ to prevent oxidation and was incubated at 4 °C. After 24 h, the solution was decanted, thereby resulting in a cold extract that was centrifuged at 8000× *g* for 10 min. The resulting supernatant was filtered using Whatman filter paper No. 2. To remove lipids from the sample, the acetone was removed under reduced pressure and methanol (50% aqueous, *v*/*v*) was added. After three consecutive hexane extractions, the extracts were lyophilized to a dry powder after removing the methanol to prevent oxidation. All of the prepared samples were stored at −80 °C until needed.

### 2.2. Analysis of Phenolic Bioactive Compounds by High-Pressure Liquid Chromatography (HPLC)

The polyphenolic bioactive compounds present in the prepared WPE were analyzed by HPLC-photodiode array (PDA) analysis using a Hitachi Primaide 1430 HPLC system (Hitachi, Milford, MA, USA) with a Waters Symmetry^®^ C18 column (3.9 × 150 mm, 5 μm inner diameter) (Waters, Milford, MA, USA). The isocratic mobile phase was composed of water/acetonitrile/acetic acid (88:10:2, *v*/*v*/*v*) and was applied at a flow rate of 0.75 mL/min for ellagic acid, (+)-catechin, chlorogenic acid, and gallic acid. These four compounds were also purchased from Sigma Aldrich (St. Loius, MO, USA) to serve as standards. The injection volume was 10 μL, the oven temperature was 20 °C, and the detection wavelength was 250 nm. The retention time of pure gallic acid, (+)-catechin, chlorogenic acid, and ellagic acid were 2.053 min, 3.900 min, 4.287 min, and 19.940 min, respectively. A standard calibration curve was generated after diluting these standards with methanol.

### 2.3. Cell Culture

The human colon cancer cell line, HCT116, was obtained from the American Type Culture Collection (ATCC, Rockville, MD, USA) and was maintained in McCoy’s 5A medium (Welgene, Daegu, Korea) containing 10% fetal bovine serum (FBS) (Hyclone, Logan, UT, USA), 100 U/mL penicillin, and 100 μg/mL streptomycin (Invitrogen, Carlsbad, CA, USA). The cells were maintained at 37 °C in a 95% air and 5% CO_2_ humidified atmosphere.

### 2.4. Human Primary Cell Isolation

Collection and processing of tissue specimens were performed with approval from the institutional review board of SMG-SNU Boramae Medical Center (IRB No.: 26-2015-42). Briefly, primary CRC specimens were obtained from patients undergoing surgical resection at SMG-SNU Boramae Medical Center (Seoul, Korea). Immediately after resection, the tumor specimens were placed in transport medium consisting of McCoy’s 5A medium supplemented with 3% penicillin/streptomycin, and 1.5 μg/mL amphotericin B (Sigma Aldrich, St. Louis, MO, USA). Within 2 h of removal, excess fat and normal tissues were removed from each specimen, and tissue fragments were washed with PBS and then finely minced. Enzymatic digestion was performed with McCoy’s 5A-based medium supplemented with 1.5 mg/mL collagenase (Thermo Fisher Scientific, Waltham, MA, USA), and 20 μg/mL hyaluronidase (Sigma Aldrich) for 1–2 h at 37 °C in a shaking incubator. To isolate single cells from the tissue fragments, the suspension was filtered through a 40-μm-pore size nylon cell strainer and washed with McCoy’s 5A medium. To remove erythrocytes and cell debris, lymphocyte centrifugation (Lymphocyte-M; Cedarlane, ON, Canada) was performed. The cells were then washed with PBS and cultured in McCoy’s 5A medium supplemented with 10% FBS, 6% penicillin/streptomycin, and 3 μg/mL amphotericin B.

### 2.5. Isolation of CSCs

Expression of the CSC markers, CD133 and CD44, were detected by fluorescence-activated cell sorting (FACS) as previously described [23]. Briefly, HCT116 cells treated with 0.05% trypsin were harvested and then were washed with phosphate-buffered saline (PBS). After being washed with FACS buffer, an Alexa Fluor 488-conjugated CD44 monoclonal antibody (Cell Signaling, Danvers, MA, USA) and a CD133 monoclonal antibody (Miltenyi Biotec, Bergisch Gladbach, Germany) were incubated with the cells in the dark at 4 °C. After 30 min, the cells were analyzed with a FACS DiVa system (BD, San Jose, CA, USA). Twenty percent of the cells with the highest and the lowest levels of fluorescence were selected as positive and negative controls, respectively.

### 2.6. Cell Proliferation Assay

To detect the growth of the isolated colon CSCs, 3-[4,5-dimethylthiazol-2-yl]-2,5 diphenyl tetrazolium bromide (MTT, Sigma-Aldrich) assays were performed. Briefly, CD133^+^CD44^+^ HCT116 cells were seeded into 96-well plates (1 × 10^2^ cells/well) and then were treated with WPE (0, 10, 20, and 40 μg/mL), as well as doses of (+)-catechin, chlorogenic acid, ellagic acid, and gallic acid that were comparable to 40 μg/mL of WPE. After 2, 4, and 6 days, the cells were washed with PBS and the medium was replaced with culture medium containing 500 μg/mL MTT. The cells were incubated for another 3 h and the supernatants were discarded. The formazan crystals were dissolved completely with the addition of 100 μL of DMSO and absorbance values were measured at 560 nm with a microplate reader (Molecular Device, Sunnyvale, CA, USA).

### 2.7. RNA Preparation and Reverse Transcriptase Polymerase Chain Reaction (RT-PCR)

Total RNA was isolated with Trizol reagent (Invitrogen), according to the manufacturer’s instructions. Using a RevertAid First Strand cDNA Synthesis Kit (Thermo Fisher Scientific), cDNA was subsequently synthesized and subjected to PCR amplification with Taq polymerase (TAKARA, Tokyo, Japan) and CD133, CD44, DLK1, Notch1, β-actin, and GAPDH primers that were purchased from Bioneer Inc. (Chungwon, South Korea). The resulting PCR products were separated on a 2% agarose gel containing ethidium bromide. Relative expression levels were analyzed by the CT method and all data were normalized to β-actin or GAPDH. The sequences of the PCR primer are listed in Table 1.

### 2.8. Western Blot Analysis

Western blot assays were performed as previously described [24,25]. Briefly, cells were washed with cold PBS, and lysed with RIPA buffer, and total protein concentrations were measured with a Bio-Rad Protein Assay Kit (Bio-Rad, Hercules, CA, USA). Denatured proteins were separated by 10% sodium dodecyl sulfate-polyacrylamide gel electrophoresis (SDS-PAGE) and then were transferred to polyvinylidene membranes (Millipore, Billerica, MA, USA) at 360 mA for 3 h at 4 °C. The membranes were subsequently blocked in 5% non-fat dried milk in Tris-buffered saline (TBS) and were incubated with primary antibodies against cytokeratin 20 (CK20, Abcam, Cambridge, MA, USA), Notch1 (Novus Biologicals, Littletown, CO, USA), β-catenin (Santa Cruz Biotechnology, Santa Cruz, CA, USA), and p-GSK3β (Cell Signaling Technology, Danvers, MA, USA) overnight. After the membranes were incubated with the appropriate secondary antibodies for 1 h, bound antibodies were visualized on X-ray film using an enhanced chemiluminescence reagent (Animal Genetics Inc., Suwon, Kyonggi-do, Korea). Detection of α-tubulin (Sigma Aldrich) was performed as a loading control.

### 2.9. Colony Formation Assay

Clonogenic assays were performed as previously described [23]. Briefly, CD133^+^CD44^+^ HCT116 cells were seeded in 6-well plates (100 cells/well) and then were treated with WPE (0, 10, 20, and 40 μg/mL), as well as concentrations of (+)-catechin, chlorogenic acid, ellagic acid, and gallic acid comparable to 40 μg/mL WPE. After 6 days, the colonies were fixed with 0.9% NaCl and were stained with crystal violet (Sigma Aldrich). Stained colonies were counted and plating efficiency was evaluated as follows: Plating efficiency = (number of colonies/number of seeded cells) × 100%.

### 2.10. Sphere Formation Assay

Sphere formation assays were performed to confirm the self-renewal capacity of colon CSCs as previously described [23]. Briefly, sphere medium was prepared with DMEM-F12 (1:1; Welgene) containing 20 ng/mL epidermal growth factor (EGF, Pepro Tech, London, UK), 40 ng/mL basic fibroblast growth factor 2 (bFGF, Pepro Tech), and 2% B27 (Invitrogen) and 6-well plates were coated with a 10% poly-(2-hydroxy- ethyl methacrylate) solution (Sigma Aldrich). After CD133^+^CD44^+^ HCT116 cells were plated in the 6-well plates (5 × 10^4^ cells/well), they were treated with WPE (0, 10, 20, and 40 μg/mL) or doses of (+)-catechin, chlorogenic acid, ellagic acid, and gallic acid that were comparable to 40 μg/mL WPE. After 6 days, the number of spheres containing more than 50 cells were counted and photographed.

### 2.11. Statistical Analyses

The results presented are the means ± standard error of the mean (SEM) for at least three independent experiments. One-way analysis of variance (ANOVA), followed by Tukey’s post-hoc test, were applied with GraphPad Prism (GraphPad Software, Inc., San Diego, CA, USA) to identify statistically significant differences between the groups. *p* values less than 0.05 were considered statistically significant.

## 3. Results

### 3.1. Phenolic Compounds Detected in WPE by HPLC

**Figure 1:**
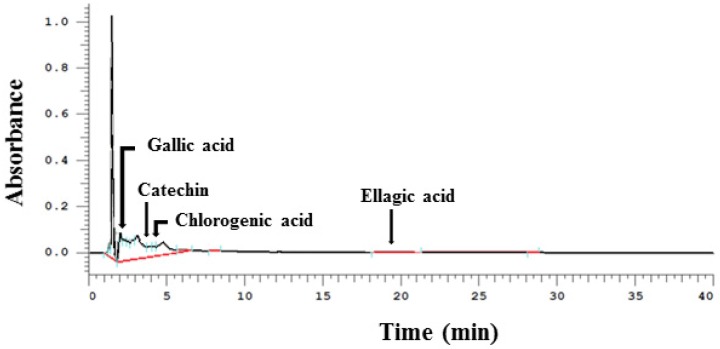
Representative HPLC chromatograms of phenolic bioactive compounds in walnut phenolic extract WPE. WPE was prepared from whole walnuts and its phenolic bioactive compounds, including gallic acid, (+)-catechin, chlorogenic acid, and ellagic, acid were detected by HPLC. WPE; walnut phenolic extract.

The major phenolic compounds that were detected by HPLC following the preparation of WPE extraction (extraction yield, 1.85%) included gallic acid, (+)-catechin, chlorogenic acid, and ellagic acid (Figure 1). Quantitative data from the HPLC analysis are presented in Table 2. In 100 g of WPE, 10.7 mg of gallic acid, 137.5 mg (+)-catechin, 13.6 mg of chlorogenic acid, and 12.6 mg of ellagic acid were detected.

### 3.2. WPE and Its Bioactive Compounds Suppress the Cell Proliferation of Colon CSCs

**Figure 2:**
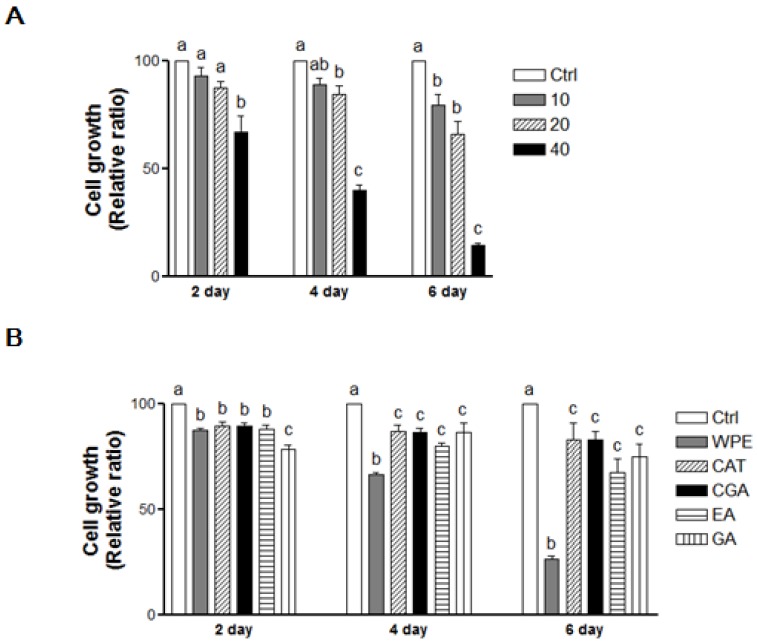
WPE and its bioactive compounds suppress the cell proliferation of colon CSCs. CD133^+^CD44^+^ HCT116 cells were treated with varying concentrations of WPE (0, 10, 20, and 40 μg/mL) (**A**); or concentrations of (+)-catechin, chlorogenic acid, ellagic acid and gallic acid comparable to 40 μg/mL of WPE (**B**). After 2, 4 and 6 days, cell growth was analyzed using MTT assays. The values shown are the means ± SEM derived from 6 replicate wells. A *p*-value < 0.05 was considered significant. Ctrl, Control; WPE, walnut phenolic extract; CSCs, cancer stem cells; CAT, (+)-catechin; CGA, chlorogenic acid; EA, ellagic acid; GA, gallic acid.

Following the treatment of CD133^+^CD44^+^ HCT116 cells with WPE (0, 10, 20, and 40 μg/mL) for 2, 4, and 6 days, cell growth was found to be suppressed in a dose-dependent manner (Figure 2A). In particular, 40 μg/mL WPE inhibited the cell growth by up to 34.4% (*p* < 0.01), 59.1% (*p* < 0.001) and 85.8% (*p* < 0.01) after 2, 4 and 6 days, respectively, compared to the control cells. Concentrations of (+)-catechin, chlorogenic acid, ellagic acid, and gallic acid that were comparable to 40 μg/mL WPE also significantly suppressed the growth of the CD133^+^CD44^+^ HCT116 cells compared to the control cells (Figure 2B). However, WPE was the most effective among these treatments at 4 and 6 days, while the individual bioactive compounds did not significantly differ in their effects on cell growth after 4 and 6 days of treatment.

### 3.3. WPE and Its Bioactive Compounds Induce the Cell Differentiation of Colon CSCs

**Figure 3:**
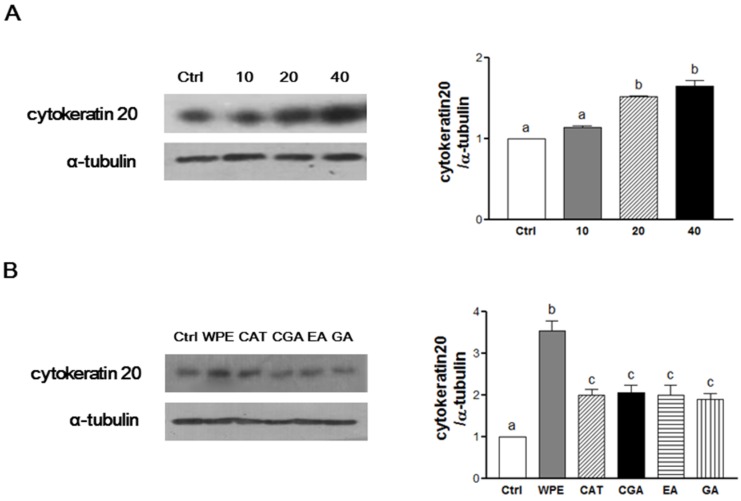
WPE and its bioactive compounds induce colon CSCs differentiation. CD133^+^CD44^+^ HCT116 cells were treated with varying concentrations of WPE (0, 10, 20, and 40 μg/mL) (**A**); or concentrations of (+)-catechin, chlorogenic acid, ellagic acid and gallic acid comparable to 40 μg/mL of WPE (**B**) for 6 days. Expressions of cytokeratin 20 (CK20) was analyzed by Western blot analysis, and α-tubulin was used as a loading control. Representative blots are shown in left panel and quantified in right panel. The values shown are the means ± SEM. A *p*-value < 0.05 was considered significant. Ctrl, Control; WPE, walnut phenolic extract; CSCs, cancer stem cells; CAT, (+)-catechin; CGA, chlorogenic acid; EA, ellagic acid; GA, gallic acid.

An important characteristic of CSCs is their ability to undergo differentiation, thereby inhibiting cell proliferation and promoting apoptosis [2]. CK20 is a differentiation marker that was significantly up-regulated following WPE treatment (Figure 3A). In particular, 40 μg/mL WPE significantly up-regulated the expression of CK20 by 164% (*p* < 0.0001) compared to the control cells. Moreover, following treatment with concentrations of (+)-catechin, chlorogenic acid, ellagic acid, and gallic acid comparable to concentrations found in 40 μg/mL of WPE, up-regulation of CK20 was also significant. However, up-regulation of CK20 by the four individual compounds did not exceed that induced by WPE (Figure 3B). Together, these results suggest that WPE and its bioactive compounds inhibit colon CSCs by inducing CSCs differentiation.

### 3.4. WPE and Its Bioactive Compounds Suppress Colon CSCs Markers, Including CD133, CD44, DLK1, and Notch1 as Well as Wnt/β-Catenin Signaling in Colon CSCs

**Figure 4:**
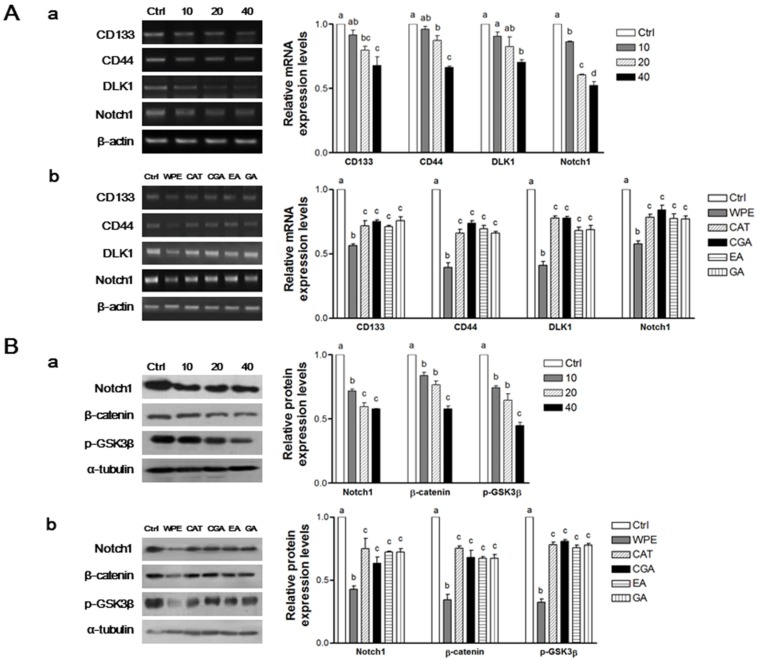
WPE and its bioactive compounds suppress colon CSCs markers, including CD133, CD44, DLK1, and Notch1 as well as Wnt/β-catenin signaling in colon CSCs. CD133^+^CD44^+^ HCT116 cells were treated with varying concentrations of WPE (0, 10, 20, and 40 μg/mL) (**Aa**,**Ba**); or concentrations of (+)-catechin, chlorogenic acid, ellagic acid and gallic acid comparable to 40 μg/mL of WPE (**Ab**,**Bb**) for 6 days. mRNA expressions of CD133, CD44, DLK1 and Notch1 were examined by RT-PCR, and β-actin was used as a loading control (**A**); Protein levels of Notch1, β-catenin and p-GSK3β were examined by Western blot analysis, and α-tubulin was used as a loading control (**B**). Representative blots are shown in left panel and quantified in right panel. The values shown are the means ± SEM. A *p*-value < 0.05 was considered significant. Ctrl, Control; WPE, walnut phenolic extract; CSCs, cancer stem cells; CAT, (+)-catechin; CGA, chlorogenic acid; EA, ellagic acid; GA, gallic acid.

To determine whether WPE inhibits the colon CSCs, mRNA levels of a panel of established CSCs markers, including CD133, CD44, DLK1, and Notch1, were investigated using RT-PCR (Figure 4A). Expression of all four CSCs markers was significantly suppressed by WPE in a dose-dependent manner (Figure 4(Aa)). Moreover, comparable doses of (+)-catechin, chlorogenic acid, ellagic acid, and gallic acid that corresponded to 40 μg/mL WPE also were highly effective in suppressing the four CSC markers. However, WPE was more effective at down-regulating the expression of these CSCs markers compared with the individual bioactive compounds (Figure 4(Ab)).

To investigate whether WPE and its bioactive compounds also inhibit the expressions of the CSCs markers, Notch1, β-catenin, and p-GSK3β, Western blot analyses were also performed (Figure 4B). The expressions of all three CSCs markers were significantly down-regulated following WPE treatment in a dose-dependent manner (Figure 4(Ba)). Furthermore, concentrations of (+)-catechin, chlorogenic acid, ellagic acid, and gallic acid that correspond to 40 μg/mL WPE also significantly down-regulated expression of the CSCs markers, although to a lesser extent than with WPE treatment (Figure 4(Bb)). These finding suggest that compounds other than the major phenolic compounds in WPE are necessary for suppressing CSCs.

### 3.5. WPE and Its Bioactive Compounds Suppress the Self-Renewal Capacity of Colon CSCs

**Figure 5:**
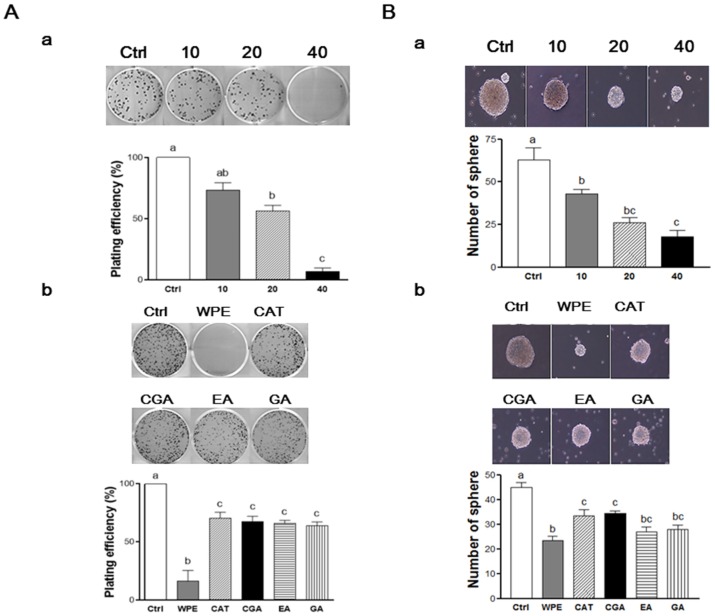
WPE and its bioactive compounds suppress self-renewal capacity of colon CSCs. CD133^+^CD44^+^ HCT116 cells were treated with varying concentrations of WPE (0, 10, 20, and 40 μg/mL) (**Aa**,**Ba**); or concentrations of (+)-catechin, chlorogenic acid, ellagic acid, and gallic acid comparable to 40 μg/mL of WPE (**Ab**,**Bb**) for 6 days. Microscopy images of colony formation were obtained (magnification, 100×, upper panel) and the number of colonies were counted (lower panel). (**A**) Sphere images were obtained using phase contrast microscopy (magnification, 100×, upper panel) and sphere numbers were counted and quantified (lower panel) (**B**). The values shown are the means ± SEM. A *p*-value < 0.05 was considered significant. Ctrl, Control; WPE, walnut phenolic extract; CSCs, cancer stem cells; CAT, (+)-catechin; CGA, chlorogenic acid; EA, ellagic acid; GA, gallic acid.

To evaluate the self-renewal capacity of CSCs, the formation of colonies from single cells were observed in colony formation assays [26]. When CD133^+^CD44^+^ HCT116 cells were treated with various concentrations of WPE, the number of cells that formed colonies decreased in a dose-dependent manner (Figure 5(Aa)). In particular, treatment of 40 μg/mL WPE suppressed the colony formation by up to 94% compared to the untreated control cells (*p* < 0.001). Concentrations of (+)-catechin, chlorogenic acid, ellagic acid, and gallic acid were comparable to 40 μg/mL WPE and significantly suppressed colony formation when compared to the untreated control cells (Figure 5(Ab)), and the extent of colony suppression was similar among the four polyphenols. However, WPE was more effective in suppressing colony formation than each of individual bioactive compounds.

To confirm the ability of WPE to mediate an effect on the anti-self-renewal property, the ability of CD133^+^CD44^+^ HCT116 cells to form non-adherent spheroids in serum-free CSCs medium was examined [27]. Following treatment with WPE (0, 10, 20, and 40 μg/mL) for 6 days, the size and number of the spheres that formed was found to decrease in a dose-dependent manner (Figure 5(Ba)). Sphere formation was suppressed by up to 72.3% compared to untreated control cells following treatment with 40 μg/mL WPE (*p* < 0.001). Doses of (+)-catechin, chlorogenic acid, ellagic acid, and gallic acid comparable to 40 μg/mL WPE also significantly suppressed the sphere formation compared to untreated control cells, with similar effects observed for all four compounds (Figure 5(Bb)). However, WPE was the most effective at inhibiting sphere formation. Taken together, these results indicate that WPE and its bioactive compounds are able to suppress CSCs by regulating their self-renewal capacity of CSCs.

### 3.6. WPE Down-Regulates CD133, CD44, DLK1, and Notch1 in Human Primary Cells Obtained from CRC Tissue

**Figure 6:**
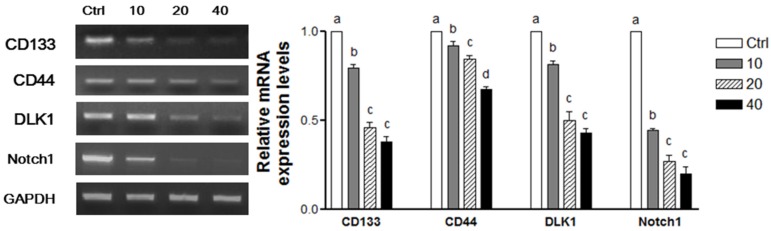
WPE down-regulates CD133, CD44, DLK1, and Notch1 in human primary cells from colorectal cancer tissue. Primary cancer cells were treated with varying concentrations of WPE (0, 10, 20, and 40 μg/mL) for 3 d. mRNA expressions of CD133, CD44, DLK1 and Notch1 were detected by RT-PCR, and GAPDH was used as a loading control. The values shown are the means ± SEM. A *p*-value < 0.05 was considered significant. Ctrl, Control.

To confirm the anti-CSCs effect of WPE, the human primary cells from human CRC tissues were isolated and then treated with varying concentrations of WPE. When the mRNA levels of CD133, CD44, DLK1, and Notch1 were subsequently analyzed (Figure 6), all four stem cell markers were found to be significantly down regulated by WPE in a dose-dependent manner. Consistent with the results obtained from the CD133^+^CD44^+^ HCT116 cells, treatment with 40 μg/mL WPE result in a 62.0%, 33.5%, 57.1%, and 81.1% decreased expression of CD133, CD44, DLK1, and Notch1, respectively, compared to the levels of each mRNA in the control cells (*p* < 0.001 in each case).

## 4. Discussion

Over the past several years, the incidence rate of CRC has decreased by more than 4% per year. However, 1 in 20 of Americans will still suffer from CRC in their lifetime [1]. Moreover, despite advances in cancer therapy, 50% of patients with CRC develop drug-resistant tumors [1], and patients with malignant CRC have a poor prognosis. As a result, there is a significant reduction in five-year relative survival rates, ranging from 92% when diagnosed at stage I to 11% at stage IV [28]. It has been shown that CSCs play an important role in tumor invasion and metastasis [2] consistent with their highly proliferative and invasive phenotype. CSCs can also increase tumor resistance to radiation and chemotherapy [2,29]. Therefore, targeting of CSCs is essential for the successful treatment of CRC in order to prevent tumor recurrence and metastasis.

Of the CSCs surface markers have previously been characterized, CD133 and CD44 were used in the present study. CD133 (prominin-1) was the first identified based on its expression by colon CSCs in immune deficient mice [30]. CD133^+^ cells exhibit a more aggressive phenotype and enhanced tumorigenic characteristics compared to CD133^−^ cells [30,31]. Moreover, clinically, CD133^+^ tumors have been shown to induce resistance to chemotherapy and are associated with poor prognosis with metastasis [32,33]. CD44 has been shown to have important roles in cancer progression, particularly in cell adhesion, invasion, and migration [34]. CD44^+^ cells have also exhibited a strong colony forming capacity and tumor growth in xenograft mouse models compared to CD44^−^ cells [35]. Previous studies have demonstrated that the detection of at least two CSCs markers is more reliable for the identification of CSC populations in human CRC. For example, CD133^+^CD44^+^ populations have been found to exhibit a tumorigenic phenotype, while CD133^−^CD44^+^, CD133^+^CD44^−^, and CD133^−^CD44^−^ populations do not [36]. Correspondingly, 1 × 10^4^ CD133^+^CD44^+^ cells have been sufficient to establish a tumor in a xenograft mouse model [23]. Thus, CD133^+^CD44^+^ cells were isolated in the present study to represent colon CSCs.

Differentiation is a characteristics of CSCs, and it involves blockade of replication and the development of specialized cells [29]. Thus, induction of differentiation in CSCs potentially represents an effective means of suppressing cancer stemness. In the present study, WPE and its bioactive compounds induced the differentiation of CD133^+^CD44^+^ HCT116 cells by up-regulating CK20. Cytokeratins are intermediate filament proteins and CK20 has previously been used as a differentiation marker of normal epithelium and CRC [37]. Several studies have demonstrated that dietary components and polyphenols can induce the differentiation of CRCs. For example, eicosapentaenoic acid (EPA), has been shown to induce the up-regulation of CK20 expression and to reduce the expression of CD133 in colon CSCs [38]. Curcumin, ellagic acid, gallic acid, quercetin, and resveratrol have also been shown to induce differentiation in CRCs [39]. Furthermore, the polyphenols in *Sasa quelpaertensis* leaf extracts (including *p*-coumaric acid and tricin) have suppressed colon CSCs by inducing their differentiation [23].

The strong expression of stem cell markers is another important CSC characteristic. Up-regulated expression of DLK1/Notch1 has been detected in various human cancers [23,40]. Aberrant Wnt/β-catenin signaling also plays a critical role in colon CSCs [4]. Thus, regulating the expression of stem cell markers could affect CSCs maintenance. In the present study, expression of DLK1, Notch1, and β-catenin/p-GSK-3β were down-regulated by WPE and its bioactive compounds. Furthermore, aberrant CSCs signaling pathways have been shown to enhance the self-renewal capacity of CSCs [4]. In the present study, the self-renewal capacity of the isolated CD133^+^CD44^+^ HCT116 cells was suppressed by WPE and its bioactive compounds in both clonogenic assays and sphere formation assays. Taken together, these results indicated that WPE and its bioactive compounds are able to suppress colon cancer stemness by regulating cell surface marker expression, signaling pathways, and self-renewal capacity.

Numerous studies have demonstrated that polyphenols in walnuts can mediate an anti-colon cancer effect. Epigallocatechin gallate (EGCG), a major catechin in green tea, has been shown to decrease the number of aberrant crypt foci and reduce the accumulation of β-catenin in premalignant lesions of azoxymethane-treated mice [41]. Ellagic acid and gallic acid in walnut extracts have also been reported to mediate antiproliferative activity in Caco2 cells [22]. In SW480 colon cancer cells, quercetin has inhibited expressions of the Wnt/β-catenin signaling pathway [42]. More recently, several studies have found that a number of individual bioactive components, including curcumin, sulforaphane, and piperine, can affect the self-renew capacity of CSCs [43,44].

In the present study, WPE was more effective inducing anti-CSCs effects in CD133^+^CD44^+^ HCT116 cells compared to individual bioactive compounds present in WPE. This may be due to the additive or synergistic effects of the individual compounds in WPE in regulating colon CSCs. In the present study, (+)-catechin, chlorogenic acid, ellagic acid, and gallic acid were detected as major compounds in WPE and each individual compound showed less anti-CSCs effect compared with WPE. It has been reported that walnut pellicle and kernel contain a number of bioactive compounds, including caffeic acid, juglone, and *p*-coumaric acid in addition to the major compounds we analyzed [45]. The anti-cancer effect of EGCG, ellagic acid, and gallic acid [41,46] and Juglone [47,48] has been reported in vitro and in vivo. In addition, caffeic acid and coumaric acid derivatives have been shown to suppress the growth of colon cancer cells by regulating apoptosis [49,50]. In the present study, various bioactive compounds and their derivatives could not be comprehensively analyzed due to limitations of the HPLC system. Therefore, there is a possibility that the combination of various compounds in WPE was more effective against cancer, rather than the individual compounds.

Although it has been demonstrated that each single nutrient or bioactive component of the food is effective for disease prevention, interactions between the different components within the food may play an important role of the whole foods. Recently, it has been an increased interest in whole-food synergy, which hypothesizes that a combination of nutrients or bioactive components increases the disease preventive effect, compared to an isolated compound. It has been reported that a combined treatment of *p*-coumaric acid and tricin showed less effect on the inhibition of self-renewal capacity and stem cell marker expression than *Sasa quelpaertensis* leaf extract in colon CSCs [23]. Shi et al. has reported that lycopene and other carotenoids from tomatoes were synergistic in anti-oxidant properties compared with individual carotenoid properties [51]. However, a limited number of studies have investigated the anti-CSCs effects of individual compounds or their combinations. Further research is required to examine the effects of two or several combination of bioactive compounds on CSCs and to compare their effects with WPE to understand their interactive effects on CSCs.

Cancer cell lines are widely used to study various aspects of tumor cell biology; however, it remains uncertain whether established cancer cell lines truly represent the characteristics of the original cancer from which they are derived. Moreover, while primary cancers grow in a partially hypoxic three-dimensional environment, cancer cell lines are generally grown in an artificial environment [52]. These differences can lead to inherent genomic and transcriptional differences, as well as phenotype and behavioral differences between cancer cell lines and tumor samples. In the present study, we examined the effect of WPE on the stemness of primary colon cancer cells. Consistent with our findings in CD133^+^CD44 HCT116 cells, we found that WPE treatment of primary cancer cells isolated from CRC tissue resulted in similar changes. These results further support the usefulness of CD133^+^CD44 HCT116 cells as representative of colon CSCs, providing a reasonable in vitro model to investigate the CSCs stemness.

## 5. Conclusions

To our knowledge, these are the first reported results to demonstrate the ability of WPE and its bioactive compounds to mediate an inhibition of colon CSCs by inducing cell differentiation, down-regulation in the expression of the CSC markers, CD133, CD44, DLK1, Notch1, and Wnt/β-catenin signaling pathways, and suppress CSC self-renewal capacity. Together, these results suggest that WPE has the potential to prevent and treat human malignant CRC by regulating CSCs, and further studies are warranted.

## Figures and Tables

**Table 1:** Primers for reverse transcriptase polymerase chain reaction RT-PCR.

Target	Forward Primer (5′ →3′)	Reverse Primer (3′ →5′)
CD133	TGGATGCAGAACTTGACAACGT	ATACCTGCTACGACAGTCGTGGT
CD44	CCAATGCCTTTGATGGACC	TCTGTCTGTGCTGTCGGTGAT
DLK1	CTGAAGGTGTCCATGAAAGAG	GCTGAAGGTGGTCATGTCGAT
Notch1	GAGGCGTGGCAGACTATGC	CTTGTACTCCGTCAGCGTGA
β-actin	ATTGGCAATGAGCGGTTC	GGATGCCACAGGACTCCAT
GAPDH	AGAAGGCTGGGGCTCATTTG	AGGGGCCATCCACAGTCTTC

**Table 2:** Quantitative determination of HPLC analysis on phenolic compounds present in phenol extract of walnut (WPE).

Phenolic Compounds	Concentration (mg/100 g of WPE)
Gallic acid	10.7
(+)-Catechin	137.5
Chlorogenic acid	13.6
Ellagic acid	12.6

Values are mean (*n* = 3).
